# Affective Instability, Depression, and Anxiety Symptoms in a Community Sample of Pregnant and Postpartum Women: A Cross-Sectional Study

**DOI:** 10.3390/ijerph19063171

**Published:** 2022-03-08

**Authors:** Hua Li

**Affiliations:** College of Nursing, University of Saskatchewan, Health Sciences Building, E-Wing, Room 4248, 104 Clinic Place, Saskatoon, SK S7N 2Z4, Canada; hua.li@usask.ca; Tel.: +1-(306)-966-2158; Fax: +1-(306) 966-6621

**Keywords:** affective instability, depression, anxiety, perinatal women

## Abstract

Background: Although perinatal women experience an elevated level of affective instability (AI), limited research has been conducted to examine perinatal AI and its relation to depression and anxiety. The current study investigated correlations between AI and depression, between AI and anxiety during the perinatal period, and between current depression and anxiety and the latent factors of the Affective Lability Scale-18 (ALS-18). Methods: 202 Canadian perinatal women participated in this cross-sectional study. The ALS-18 was used to assess AI, while the Depression, Anxiety, and Stress Scale-21 measured depression, anxiety, and stress. Multiple logistic regression was performed to investigate the relationship between AI and depression and anxiety, and multiple linear regression was conducted to examine the association between current depression and anxiety and the three latent factors of ALS-18. Results: The findings revealed a significant association between AI and depression and between AI and anxiety. Current depression and anxiety were correlated with ALS-18 factors of depression/anxiety shift and depression/elation shift, while current depression was linked to ALS-18 factor of anger. Conclusions: The study findings have important implications for early detection and intervention of maternal anxiety and depression. In order to improve maternal mental health, AI should be included in routine perinatal check-ups.

## 1. Introduction

The transition to motherhood often represents a period where women face increased vulnerability to developing anxiety and mood-related disturbances, including depression and affective instability (AI) [1,2,3]. These mood-related symptoms and anxiety are thought to be caused by hormonal fluctuations that occur during this period of time [4,5]. In the past several decades, depression and anxiety during pregnancy and postpartum have been researched extensively because of their possible adverse effects on mothers and their offspring’s physical and mental health [6,7,8,9]. For example, during the perinatal period, women who experience depressive and/or anxiety symptoms are more likely to experience somatic symptoms and withdrawal, use substances, experience complications during labor/birth, and less likely to attend perinatal classes and routine check-ups and adhere to a nutritious diet [6,10,11,12,13,14,15]. Studies show that mothers with perinatal depression are at a significantly higher risk for suicidality [7,16]. In addition, antenatal depression and anxiety have been associated with adverse neonatal outcomes such as low BW, small for gestational age (SGA), preterm birth, and low Apgar score [17,18,19,20]. Significant associations between postpartum depression and internalizing and externalizing psychopathologies in children have been documented in a meta-analysis study [21], while antenatal depression has been linked with an increase in child externalizing difficulties and a decrease in cognitive function [22]. Antenatal anxiety is positively linked with behavior and emotional problems in children [23], and postpartum anxiety has negative effects on children’s temperament, sleep, and cognitive development [24]. However, AI has been largely neglected, even though mood fluctuations are a prominent feature in pregnant and postpartum women [25].

Affective instability is defined as sudden and extreme mood fluctuations in an individual over time [26]. Due to a lack of agreement on the definition, AI is sometimes used interchangeably with mood dysregulation, affective lability, emotional dysregulation, and mood swings [27]. As a transdiagnostic concept in mental health, the prevalence of AI in the adult population is estimated at 13.9% based on the Psychiatric Morbidity Survey in the UK [28]. Emotional experiences including negative affects and neuroticism have been associated with AI according to psychological functioning studies [29,30], while the literature supports the notion that AI is correlated with psychopathologies in the general population and clinical samples including anxiety and depression [31]. AI has also been identified as an independent predictive factor in explaining suicidal thoughts in population studies [32,33].

Affective instability has been known as a part of women’s life during their reproductive years, especially during pregnancy and postpartum [34,35]. Based on the literature [8,36,37], AI in perinatal women has been investigated largely under the concept of postpartum blues, a time frame of 14 days after giving birth, while AI as a distinct feature across pregnancy and postpartum period has been less studied. In a longitudinal study, Bowen A. et al. [25] found that perinatal women experience a significantly higher level of AI at early and late pregnancy and postpartum compared to non-perinatal women with normal menstrual cycles. A recent study investigated the relationship between AI and depression and showed that perinatal AI was strongly correlated with depression at early and late pregnancy and postpartum cross-sectionally, while AI at early pregnancy was significantly associated with postpartum depression prospectively while controlling baseline depression [38], which is in agreement with previous studies in non-perinatal women populations [39,40]. Although previous evidence suggests that AI is linked to depression among perinatal women, few studies have been conducted, especially using validated instruments to measure AI, such as Affective Lability Scale-18 [41], Affective Control Scale [42].

The current study aimed to investigate the associations between AI and depression and between AI and anxiety after adjusting for other confounders in a community sample of pregnant and postpartum women.

## 2. Materials and Methods

### 2.1. Participants and Procedure

A total of 202 pregnant and postpartum women participated in this cross-sectional study, and they came from nine different provinces in Canada through an online survey from April 2020 to October 2020. Advertisement of recruitment information was posted on several reputable Canadian maternal websites (e.g., This West Coast Mommy, Ooh! Motherhood). Inclusion criteria included Canadian women who were English-speaking, ≥18 years old, and pregnant or within 12 months of postpartum. Ethics approval was obtained from the Behavioural Research Ethics Board of the University of Saskatchewan. Online informed consent was obtained from all participants. All participating women received an honorarium of 10 Canadian dollar e-gift card.

### 2.2. Measures

**Affective instability**. AI was measured by using the Affective Lability Scale-18, an 18-item self-reported questionnaire [41]. The respondents rated their AI symptoms on a 4-point (0–3) scale ranging from “very uncharacteristic of me” to “very characteristic of me” with a maximum score of 54. Factor analysis studies on three latent factors of the ALS-18 suggested a good fit: anxiety/depression shift (items 1, 3, 5, 6, and 7), depression/elation shift (items 2, 10, 12, 13, 15, 16, 17, and 18), and anger (items 4, 8, 9, 11, and 14) [41]. Psychometric properties of ALS-18 have been examined in different populations, including in patients with personality disorders, ADHD, bipolar disorder, and in perinatal women with mood symptoms, and the studies recommended the ALS-18 as a valid instrument for assessing AI [43,44,45,46]. The cutoff score for AI symptoms was based on the mean of ALS-18 (>mean vs. ≤mean).

**Depression,anxiety, and stress**. The Depression, Anxiety, and Stress Scale -21 (DASS-21) was used to assess depression, anxiety, and stress among participating women [47]. This 21-item self-report questionnaire includes three subscales: depression (items 3, 5, 10, 13, 16, 17, and 21), anxiety (items 2, 4 7, 9, 15, 19, and 20) and stress (items 1, 6, 8, 11, 12, 14, and 18) [47]. The respondents rated each item in the past week ranging from 0 (did not apply to me at all) to 3 (applied to me very much). To be compatible with the DASS-42 cut-offs, the DASS-21 score needs to be multiplied by 2 [47]. Cutoff scores for clinically significant symptoms are suggested to be 60 for the total score, while cutoff scores for subscales of depression, anxiety, and stress to be 21, 15, and 26, respectively [48].

As a measure of three distinct constructs, DASS-21 was an appropriate instrument for the current study for the following reasons. First, the psychometric properties of the instrument are well-established in assessing depression, anxiety, and stress in clinical and nonclinical populations [47,49,50,51], including perinatal women [52,53]. A high level of internal consistency (Cronbach’s α) was found for all subscales: 0.84 for depression, 0.77 for anxiety, and 0.86 for stress in postpartum women [54]. In terms of DASS-21 total, internal consistency of 0.74 in pregnancy and 0.79 in postpartum was reported [55].

Secondly, with regard to convergent validity, the DASS-21 depression subscale is highly positively correlated with EPDS (r = 0.84) and with Beck Depression Inventory (BDI-II, r = 0.82), while the DASS-21 anxiety subscale is strongly correlated with the Beck Anxiety Inventory (r = 0.86) [56]. DASS-21 is frequently used to measure depression and anxiety among perinatal women [55,57,58,59].

**Social support**. The multidimensional Scale of Perceived Social Support (MSPSS) was used to measure social support among participating women [60]. MSPSS is a self-reported scale with 12 items that assesses the perceived social support that an individual receives from three sources: friends (items 6, 7, 9, and 12), family (items 3, 4, 8, and 11), and significant other (items 1, 2, 5, and 10) [60]. Each item is rated on a seven-point Likert scale with responses ranging from very strongly disagree (=1) to very strongly agree (=7) with a total possible score of 84. MSPSS show high internal reliability of Cronbach’s α 0.88 for the total score, and 0.91, 0.87, and 0.85 for the subscales of significant other, family, and friends, respectively [60], while in another study, Cronbach’s α for the three subscales were 0.85, 0.90, and 0.88, respectively, and for the total scale it was 0.87 [61]. MSPSS has been used in assessing social support among perinatal women [62,63]. The cutoff score for social support was based on the mean of MSPPS, and MSPPS subscales of significant other, family, and friends (>mean vs. ≤mean).

Other information of participating women was also collected including sociodemographic variables, history of depression and/or anxiety, family history of mental illness, complications during pregnancy or labor/birth, preterm birth, and baby’s overall health.

### 2.3. Data Analyses

#### 2.3.1. Descriptive Statistics

Descriptive statistical analysis was conducted to summarize the characteristics of the participants. The percentages of women who scored above the mean of ALS-18, above or equal to the thresholds of depression, anxiety, and stress DASS-21 subscales, and above the mean of MSPPS were calculated. Group-specific descriptive statistical analyses were also performed to describe AI levels in all women and pregnant and postpartum women. Cronbach’s α was calculated to assess internal reliability for all measures.

#### 2.3.2. Group Comparisons

Two-sample t-test was used to compare the mean of AI between women with ≥21 on the DASS-21 depression subscale and women with <21 on the DASS-21 depression subscale, between women with ≥15 on the DASS-21 anxiety subscale and women with <15 on the DASS-21 anxiety subscale, between women with ≥26 on the DASS-21 stress subscale and women with <26 on the DASS-21 stress subscale, and between women who scored higher than the mean on MSPPS and women who scored below or equal to the mean on MSPPS.

#### 2.3.3. The Correlation of Perinatal AI with Depression and Anxiety

To investigate the association between AI and depression and between AI and anxiety, binary logistic regression analysis was used to generate univariate odds ratios associated with each independent variable, including depression, anxiety, sociodemographic variables (marital status, ethnicity, educational attainment, family financial situation), history of depression, history of anxiety, family history of mental illness, stress, social support, complications during pregnancy or labor/birth, preterm birth, and baby’s overall health. Multiple logistic regression was employed for variables with *p* ≤ 0.25 in univariate analyses.

The ALS-18 is rested on three dimensions: anxiety/depression shift, depression/elation shift, and anger [41]. We conducted linear regression analysis to examine the relationship between each dimension of ALS-18 and current depression or anxiety.

The results were considered to provide evidence of significance if *p*-values < 0.05 (α level of 5%). The data were analyzed using SPSS 27.

## 3. Results

### 3.1. Characteristics of Participating Women

A total of 202 perinatal women participated in the study, including 94 pregnant women with a mean of 23.6 weeks of pregnancy (*SE* = 10.04) and 108 postpartum women with a mean of 24.4 weeks of postpartum (*SE* = 15.03). Table 1 presents the characteristics of the participating women.

### 3.2. Psychosocial Characteristics of Participating Women and Group Comparisons

Affective instability was common, present in 50% of the participating women (ALS-18 score > mean), while 24.3% of women were screened positive for depression (DASS-21 depression subscale score ≥ 21), 28.7% had a score for anxiety above the threshold (DASS-21 anxiety subscale score ≥ 15), 37.1% felt stressed (DASS-21 stress subscale score ≥ 26), and 38.1% reported a lack of social support (≤ mean of MSPSS score) (Table 2).

The means and standard deviations of ALS-18 score, DASS-21 total and subscales scores, MSPSS total and subscales scores, and percentage of women who scored above or equal to the threshold of all measures are presented in Table 2. The results of Cronbach’s α (ranging from 0.805 to 0.936) indicated high internal consistency for all measures (Table 2).

When comparing groups of women who scored above or equal to the threshold of each measure and who scored below the threshold of each measure (AI, depression, anxiety, or stress), the results of two-sample t-test statistics with the degree of freedom showed that women who scored above or equal to the threshold of each measure experienced a significantly higher level of AI (ALS-18 score: t(197) = 20.44, *p* < 0.001), a higher level of depression (DASS-21 depression subscale: t(54) = 16.43, *p* < 0.001), a higher level of anxiety (DASS-21 anxiety subscale: t(62) = 14.15, *p* < 0.001), and a higher level of stress (DASS-21 stress subscale: t(119) = 18.36, *p* < 0.001). Women who reported a lack of social support showed a significantly elevated level of AI in comparison with that of their counterparts (MSPSS: t(98) = 15.30, *p* < 0.001).

**Figure 1:**
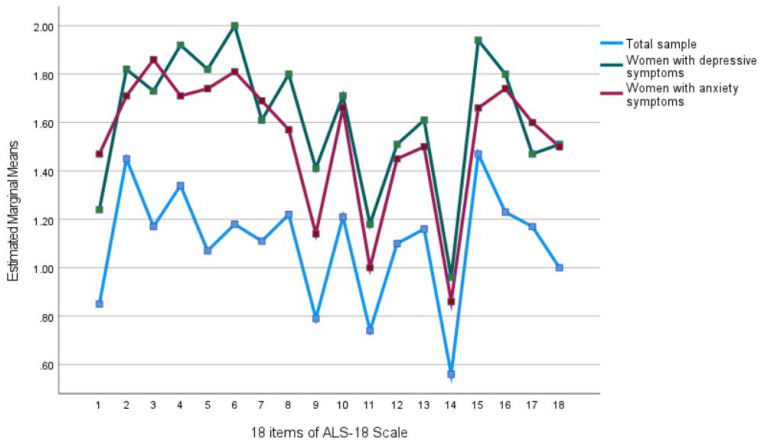
The cross-sectional association of perinatal affective instability, depression, and anxiety.

For the level of AI in different group comparisons, the AI level in women who scored above or equal to the threshold of each measure and those who scored below the threshold of each measure (depression, anxiety, stress, or lack of social support) displayed significant group differences (depression: t(97) = 8.17, *p* < 0.001; anxiety: t(116) = 7.29, *p* < 0.001; stress: t(163) = 9.390, *p* < 0.001; social support: t(174) = 6.82, *p* < 0.001, respectively). Means on each item of ALS-18 among all participating women, women with depression symptoms, and women with anxiety symptoms are presented in Figure 1.

When comparing pregnant and postpartum women, there were no significant group differences in AI (t(197) = 0.11, *p* = 0.913), depression (t(193) = 0.579, *p* = 0.564), anxiety (t(164) = 1.78, *p* = 0.077), stress (t(194) = 0.087, *p* = 0.931), and social support (t(200) = 0.82, *p* = 0.412).

### 3.3. The Association between AI and Depression and Anxiety

A majority of independent variables showed a statistically significant correlation with AI in univariate logistic regression (Supplement Table S1). Multivariate logistic regression results revealed that depression, anxiety, history of depression, history of anxiety, and support from significant others were strongly linked to AI (Table 3), indicating significant independent effects, while holding all other variables constant. Estimated ORs from the model indicated that women with scores above or equal to the threshold for depression and anxiety were 3.87 times and 2.97 times more likely to experience AI symptoms, respectively (Table 3). Even though stress and social support from family and friends were found to be significantly associated with AI in univariate analysis, they were not established as independently operating risk factors in multivariate logistic regression analysis.

History of depression and history of anxiety were strongly associated with current AI. To assess whether the associations were due to including women who experienced depression and/or anxiety currently, we examined the associations by excluding women with current depression and/or anxiety, respectively, and the results showed that history of depression and history of anxiety were still significantly linked with current AI (t(151) = 3.57, *p* = 0.001; t(142) = 3.44, *p* < 0.001 respectively).

Current depression and anxiety were significantly associated with the ALS-18 factor of depression/anxiety (affect shift between depression and anxiety, estimate coefficient for depression and anxiety: 2.315 and 3.454, respectively) and with the ALS-18 factor depression/elation (affect shift between depression and elation, estimate coefficient for depression and anxiety: 3.392 and 2.777, respectively), while depression was linked to the ALS-18 factor of anger (estimate coefficient for depression: 2.979) (Table 4).

## 4. Discussion

The first main finding of this study was that perinatal AI was strongly and independently associated with depression and anxiety. Our findings replicate those of previous work in general and clinical populations [40,64] and confirm the relationship between AI and depression in perinatal women [38] while filling the research gap of associations between AI and anxiety and between a history of anxiety and AI in pregnant and postpartum women. It is the first known study using a validated instrument to examine AI in perinatal women and its relations to depression and anxiety.

Previous research found that history of depression or history of anxiety are identified as the strongest predictors for current depression or anxiety, respectively, in both perinatal and non-perinatal populations [65,66,67]. Studies elucidating the correlations largely under the stress causation theory suggest that even after a depressive or anxiety episode, a heightened level of stress, including interpersonal stress and non-interpersonal chronic life stress, continues to be maintained for a period of time in individuals with depressive and/or anxiety disorders [68,69], while neuroticism as a shared property between depression, anxiety, and AI, partially accounts for the associations [69]. In the current study, a history of depression or a history of anxiety was linked to current AI while excluding women with current depression or anxiety, indicating that a history of depression or a history of anxiety independently predict current AI, which may be explained by the stress causation theory.

Notably, current depression and anxiety were significantly associated with ALS-18 factors of depression/anxiety shift and depression/elation shift. The associations may be elucidated by the high comorbidity between depression and anxiety [70], hypomania as a core symptom of depression [71], and strong genetic correlations between AI, depression, and anxiety [72]. The findings are also in agreement with those of a previous study that suggested that affective experiences in perinatal women can be depicted in a labile fashion [43]. Additionally, depression was linked with the ALS-18 factor of anger in this study, which confirmed the notion that anger is a prominent feature of depression and plays an integral role in the development of depression [73].

As a transdiagnostic concept, AI and its relationship with depression and anxiety have been illustrated as sharing some correlates, including neuroticism, negative affect, stress, and emotional awareness [74,75]. Furthermore, there is evidence of a genetic association between AI, depression, and anxiety [72]. Neuroticism, negative affect, and stress as shared properties among AI, depression, and anxiety have been discussed in a previous study [76]. Empirical research implicated the relationship between emotional awareness, emotion regulation, and psychopathologies including depression and anxiety by elucidating that emotional awareness (i.e., attending to and understanding one’s own emotion) is essential for effective emotion regulation [77], while emotional awareness has been consistently recognized as a strong predictor for an array of psychopathologies via associations with emotion dysregulation [78]. Studies have further explained the relationship by suggesting that emotional awareness is associated with the implementation of emotion regulation strategies [79,80]. For example, an individual who understands his/her emotion clearly is more likely to effectively utilize emotion regulation strategies including cognitive reappraisal, expressive suppression, and nonjudgmental acceptance of emotion [80]. Thus, emotional awareness and emotion regulation together contribute to psychopathology including depression and anxiety. For genetic evidence, researchers in the UK conducted a genome-wide study of mood instability in 53,525 cases and 60,443 controls from Biobank and found a significant genetic correlation between mood instability, depression, anxiety, and schizophrenia [72].

Lack of social support from significant others was identified as a risk factor for AI in this study. During the perinatal period, women experience tremendous changes physically and psychologically which require major adjustments, and social support plays a crucial role to help women cope with the transition to motherhood [81,82]. Lack of social support from partners, family, and/or friends can be reflected in a lack of social stability and social participation, which has a negative impact on the psychological wellbeing of perinatal women, including experiencing stress [83,84]. While there is limited study on the correlation between AI and social support, the significant link found in this study may be explained by the evidence of the relationship between AI and stress in previous research. For example, a bidirectional relationship between AI and stress has been suggested [85]. Mood fluctuations may be the result of life stresses, while frequent mood shifts may cause stressful events to occur (e.g., job loss, interpersonal relationship difficulties).

There are some implications from this study. Given the evidence of the strong association between AI and depression and between AI and anxiety in perinatal women, expanding the view of perinatal mood symptoms could help understand perinatal women’s affective experience in a more comprehensive way, particularly for healthcare providers who most likely pay attention to diagnosable entities such as depression and anxiety when evaluating mood and anxiety symptoms in perinatal women. From early detection and early intervention perspectives, perinatal routine check-ups should include AI assessment that would provide another opportunity to identify women who are at risk for developing depression and/or anxiety, including women who have a history of depression and/or anxiety. In addition, the strong association between social support and perinatal depression and anxiety has been well-established [86], while the new evidence in our study revealed the significant relationship between social support and AI in perinatal women. Thus, our finding further supports the recommendation that social support should be a targeted risk factor for preventing the development of mood and anxiety symptoms or alleviating the symptoms during the perinatal period. Furthermore, interpersonal violence during the perinatal period and its negative impact on maternal mental health have been documented [87]. However, there are very limited studies on the topic of the association between interpersonal violence and AI in pregnant and postpartum women. Future research is required to explore the relation of perinatal AI with interpersonal violence.

There are several limitations in this study, including possible misrepresentations of online recruitment methods, and the composition of participants (e.g., predominantly Caucasians among study participants, self-selected sample). As a property of AI, temporality indicates that the experience of AI may change throughout the day, thus a cross-sectional design of the current study may not capture AI experience accurately. A detailed description of the limitation was discussed in a previous publication (Li et al., 2021). In addition, the impact of COVID-19 on mental health and psychological wellbeing has been reported [88,89,90]. The pandemic might have effects on participating women’s mood, anxiety, and other emotional experiences, which was not included in the data collection of this present study. Therefore, the findings of this study must be interpreted with caution.

## 5. Conclusions

The findings of this current study showed strong independent associations between perinatal AI and depression and between perinatal AI and anxiety and revealed that women’s affective experiences can be depicted in a labile fashion during the perinatal period. Thus, in order to improve maternal mental health, AI should be included in perinatal affective experiences research and clinical practice. More studies are required in this under-researched area.

## Figures and Tables

**Table 1:** Characteristics of participating women (*n* = 202).

Variables	*N* (%)
Maternal status	
Pregnant	94 (46.5)
First trimester	18 (19.2)
Second trimester	33 (35.1)
Third trimester	43 (45.7)
Postpartum	108 (53.5)
4 weeks	9 (8.3)
Week 5–6 months	56 (51.9)
7–12 months	43 (39.8)
Live with partner	
Yes	192 (95.0)
No	8 (4.0)
Missing	2 (1.0)
Educational Level	
High school	17 (8.4)
Post-secondary	57 (28.2)
University	128 (63.4)
Financial situation	
Not good	7 (3.5)
Average	108 (53.4)
Good	86 (42.6)
Missing	1 (0.5)
Ethnicity	
Caucasian	166 (79.2)
Asian	19 (9.4)
Indigenous	7 (3.5)
Hispanic/Latino	7 (3.5)
Not specified	9 (4.4)

**Table 2:** Mean scores of ALS-18, DASS-21 (total and subscales), MSPSS (total and subscales), and results of Cronbach’s α in all participating women (*n* = 202).

Measurements	Mean	*SD*	Scores ≥ Cut-Off (%)	Cronbach’s α
ALS-18 score	19.82	11.28		0.926
ALS-18 Total > mean	29.07	6.76	50.00	
ALS-18 Total ≤ mean	10.56	6.09		
DASS-21 Total ≥ 60	35.00	8.11		
DASS-21 Total < 60	18.42	10.49		
DASS-21 Depression Subscale ≥ 21	29.04	8.60		
DASS-21 Depression Subscale < 21	16.86	10.42		
DASS-21 Anxiety Subscale ≥ 15	27.66	9.40		
DASS-21 Anxiety Subscale ≥ 15	16.66	10.43		
DASS-21 Stress Subscale ≥ 26	27.83	9.09		
DASS-21 Stress Subscale ≥ 26	15.09	9.68		
MSPSS Total > mean	25.92	9.57		
MSPSS Total ≤ mean	16.06	10.61		
DASS-21 score	26.35	19.65		0.918
DASS-21 Total ≥ 60	70.82	12.67	8.42	
DASS-21 Total < 60	22.26	14.43		
DASS-21 Depression Subscale score	16.51	16.39		0.879
DASS-21 Depression Subscale ≥ 21	41.31	13.52	24.25	
DASS-21 Depression Subscale < 21	8.58	6.01		
DASS-21 Anxiety Subscale score	11.41	13.70		0.805
DASS-21 Anxiety Subscale ≥ 15	29.10	13.10	28.71	
DASS-21 Anxiety Subscale ≥ 15	4.28	4.14		
DASS-21 Stress Subscale score	23.47	16.75		0.840
DASS-21 Stress Subscale ≥ 26	40.16	10.99	37.13	
DASS-21 Stress Subscale ≥ 26	13.61	7.81		
MSPSS total score	68.01	12.87		0.936
MSPSS score ≤ mean	55.21	11.10	38.12	
MSPSS score > mean	75.90	5.33		
MSPSS significant other subscale score	24.09	4.50		0.903
Subscale score ≤ mean	20.26	4.41	43.10	
Subscale score > mean	27.00	1.18		
MSPSS family subscale score	22.52	5.13		0.912
Subscale score ≤ mean	18.61	4.66	48.51	
Subscale score > mean	26.20	1.64		
MSPSS friends subscale score	21.40	5.32		0.928
Subscale score ≤ mean	17.52	4.48	48.02	
Subscale score > mean	25.56	1.85		

**Table 3:** Multiple logistic regression results of associations between AI and depression, anxiety, and other variables (*n* = 202).

Variables	Odds Ratio (95% CI)	*p*-Value
Depression	3.874 (1.340–11.202)	0.012
Anxiety	2.973 (1.259–7.019)	0.013
History of depression	0.419 (0.186–0.942)	0.035
History of anxiety	0.275 (0.111–0.683)	0.005
Support from significant other	0.304 (0.142–0.651)	0.002

**Table 4:** Linear regression results of associations between three dimensions of ALS-18 and depression and anxiety (*n* = 202).

Variables	Estimate Coefficient (95% CI)	*p*-Value
ALS-18 factor: depression/anxiety shift		
Depression	2.315 (1.139–3.490)	<0.001
Anxiety	3.454 (2.342–4.567)	<0.001
ALS-18 factor: depression/elation shift		
Depression	3.392 (1.515–5.269)	<0.001
Anxiety	2.777 (0.999–4.555)	0.002
ALS-18 factor: anger		
Depression	2.979 (1.756–4.203)	<0.001
Anxiety	0.998 (-0.161–2.157)	0.091

## Data Availability

All data generated or analyzed during this study are included in this article including supplement table. Further enquiries can be directed to the corresponding author.

## Supplementary Materials

The following supporting information can be downloaded at: https://www.mdpi.com/article/10.3390/ijerph19063171/s1, Table S1: Univariate logistic regression results of the association between AI and other variables.
