# Supplementary material for: Affective Instability, Depression, and Anxiety Symptoms in a Community Sample of Pregnant and Postpartum Women: A Cross-Sectional Study
Source: Int J Environ Res Public Health. 2022 Mar 8;19(6):3171. doi: 10.3390/ijerph19063171 (PMC8951286; doi:10.3390/ijerph19063171)
Supplement: Supplementary file 1 [file ijerph-19-03171-s001.zip › ijerph-1434492-supplementary.pdf]

Table S1. Univariate logistic regression results of the association between AI and other variables (N = 202).

Variables	Frequency (%)	Odds ratio (95% CI)	*p* - value
Marital status		0.735 (0.293 – 1.844)	0.511
Without partner	10 (5.0)		
With partner	192 (95.0)		
Education		0.517 (0.183 – 1.456)	0.212
< high school diploma	17 (8.4)		
≥ high school diploma	185 (91.6)		
Ethnicity		0.786 (0.397 – 1.554)	0.489
Non-Caucasian	42 (20.8)		
Caucasian	160 (79.2)		
Family financial situation (missing = 8)		0.708 (0. 401 – 1.251)	0.235
Fair/poor	86 (44.3)		
Good/exellent	108 (55.7)		
DASS-21 depression scale		11.739 (4.704 – 29.293)	<.0001
Subscale score ≥ 21	49 (24.3)		
Subscale score < 21	153 (75.7)		
DASS-21 anxiety scale		6.203 (3.023 – 12.728)	<.0001
Subscale score ≥ 15	58 (28.7)		
Subscale score < 15	144 (71.3)		
Personal history of depression		0.203 (0.112 – 0.370)	<.0001
Yes	108 (53.5)		
No	94 (46.5)		
Personal history of anxiety		0.170 (0.088 – 0. 330)	<.0001
Yes	133 (65.8)		
No	69 (34.2)		
Family history of mental illness (missing = 1)		0.394 (0.215 – 0.724)	0.003
Yes	63 (31.3)		
No	138 (68.7)		
DASS-21 stress scale		5.970 (3.138 – 11.371)	<0.0001
Subscale score ≥ 26	75 (37.1)		
Subscale score < 26	127 (62.9)		
MSPSS significant other subscale		0.299 (0.166 - 0.536)	<0.0001
Subscale score ≥ mean	87 (43.1)		
Subscale score < mean	115 (56.9)		
MSPSS family subscale		0.348 (0.197 - 0.617)	<0.0001
Subscale score ≥ mean	98 (48.4)		
Subscale score < mean	104 51.5)		
MSPSS friends subscale		0.429 (0.244 - 0.755)	0.003

Subscale score ≥ mean	105 (52.0)		
Subscale score < mean	97 (48.0)		
Complications during pregnancy		0.434 (0.154 – 1.226)	0.115
Yes	19 (20.2)		
No	75 (79.8)		
Complications during labor/birth		0.820 (0.357 – 1.881)	0.638
Yes	32 (29.6)		
No	76 (70.4)		
Preterm birth (< 38 weeks)		0.597 (0.215 – 1.655)	0.638
Yes	19 (17.6)		
No	89 (82.4)		
Baby's overall health		2.245 (0.981 – 5.141)	0.056
Fair/poor	27 (25.0)		
Good/excellent	81 (75.0)		
